# Supplementary material for: Single cell electron collectors for highly efficient wiring-up electronic abiotic/biotic interfaces
Source: Nat Commun. 2020 Aug 14;11:4087. doi: 10.1038/s41467-020-17897-9 (PMC7429851; doi:10.1038/s41467-020-17897-9)
Supplement: Supplementary file 1 — Supplementary Information [file 41467_2020_17897_MOESM1_ESM.pdf]

Supplementary Information

Single cell electron collectors for highly efficient wiring-up electronic abiotic/biotic interfaces

Yu et al

Supplementary Note 1. Estimation of cell number colonized on the electrode

The number of electrode colonized cells was estimated by dividing the total protein amount of biofilm on electrode with that of a single *S. oneidensis* MR-1 (SW) cell. To determine the protein content of biofilm electrode, carbon felt electrode MFC operation was cut up, mixed with 2 mL DI water and ultrasonicated. After centrifugation, the supernatant protein content was quantified by Bradford method with bovine serum albumin serving as the standard. To obtain the protein content of single SW cell, the late logarithmic cells were harvested for total protein quantification and plate counting analyses, which disclosed that single SW cell contains 1.53×10^{-13} g protein.

Supplementary Note 2. Carbon felt electrode modification with PDA and Fe-S nanoparticle

Electrode modification was performed as the following. PDA modification was performed with electrochemical polymerization as reported previously[1]. In brief, the CF electrode was immersed in Tris-HCl buffer (10 mM, pH=8.5) and dopamine hydrochloride (4 mg mL^{-1}) was added into the solution in a three-electrode electrochemical cell (Pt counter electrode and SCE reference electrode). Then, CV scanning (-0.4 V to 0.3 V, 10 mV s^{-1}, 2 cycles) was applied for PDA polymerization on the CF electrode. After polymerization, the PDA modified electrode was rinsed three times with distilled water, dried in the air and used as MFC anode. Fe-S nanoparticle modification on CF was performed with hydro-thermo synthesis according to previous report[2] with minor modification. In brief, 5 mL 0.135 g mL^{-1} $FeCl_3$, 5 mL 0.038 g mL^{-1} $(NH_2)_2CS$ and 30 μL 28% ammonia were added into 70 mL distilled water (pH was adjusted to 2.0 with 1 M HCl). Then, 4 pieces of CF electrodes (1× 2 cm) were immersed into the solution and was heated at 180 °C for 12 h. After that, the CF electrode was rinsed three times with distilled water, dried in the air and used as MFC anode.

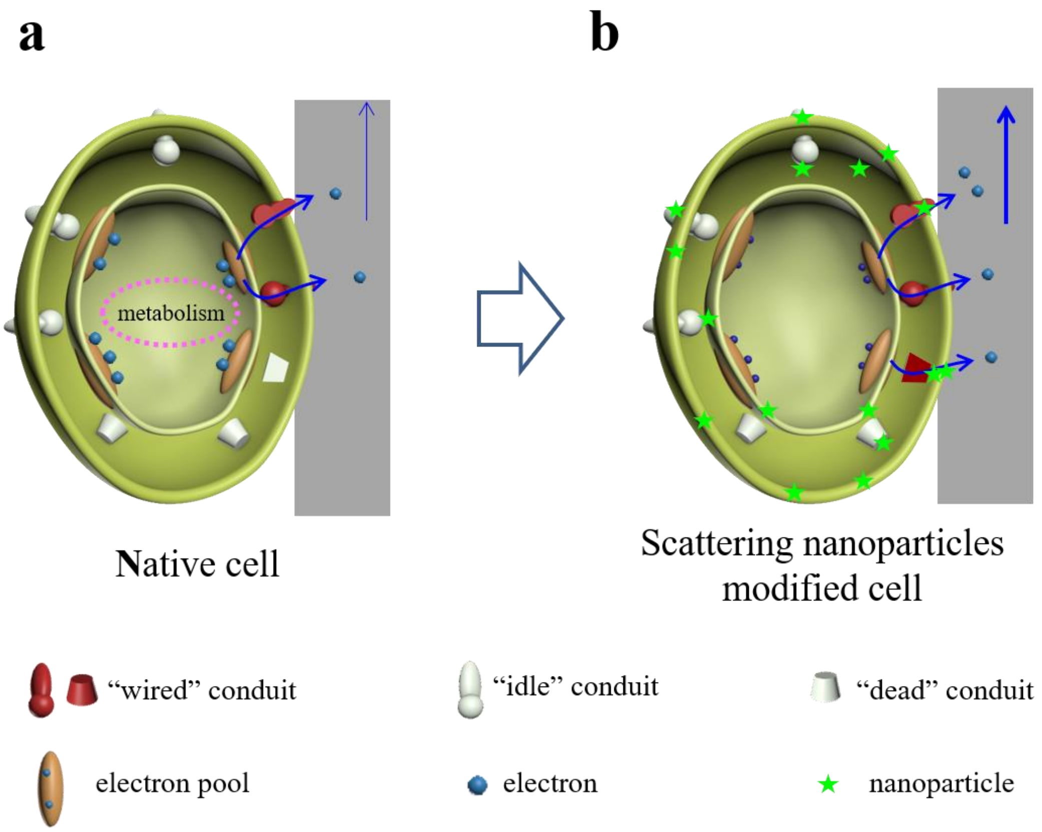

Supplementary Figure 1 | Schematic of biointerfacial electron transfer between electrode and cells. **(a)** Native cell and **(b)** Nanoparticles modified cell. The scattering nanoparticle might reinforce some "wired" conduits and activate some "dead" or "idle" conduits, but most of the "dead" and "idle" conduits were still unwired. The electron collection from individual cell still relied on the bulk electrode.

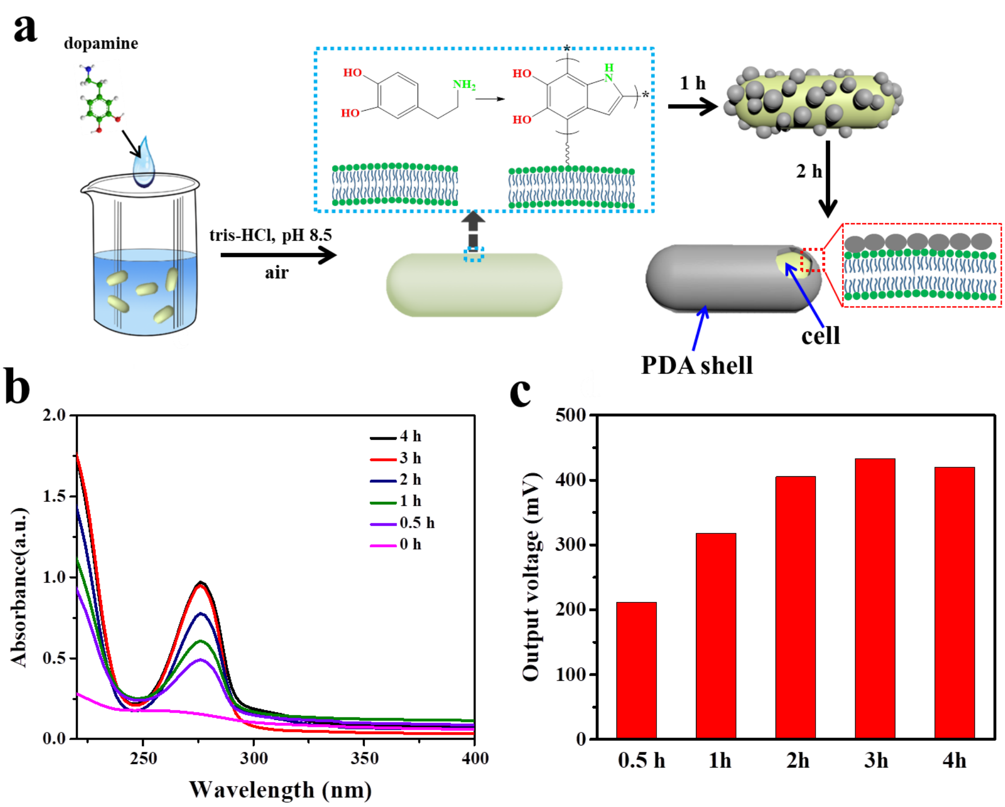

Supplementary Figure 2 | Assembly and characterization of S collector on the SW cell **(a)** the schematic illustration of *in-situ* polymerization of PDA on bacterial outer surface. **(b)** UV-vis spectrum of PDA coated SW cells with different polymerization time. Cell density was diluted to OD_{600}=0.04 for measurement. The peak around 275 nm belonged to the absorption of 5,6-dihydroxyindole, the repeating unit of PDA; **(c)** maximum output voltage of MFC inoculated with PDA coated SW cells at different polymerization period. MFC were set up with a carbon felt anode (1 cm × 2 cm) and continuously discharged with 2000 Ω resistor.

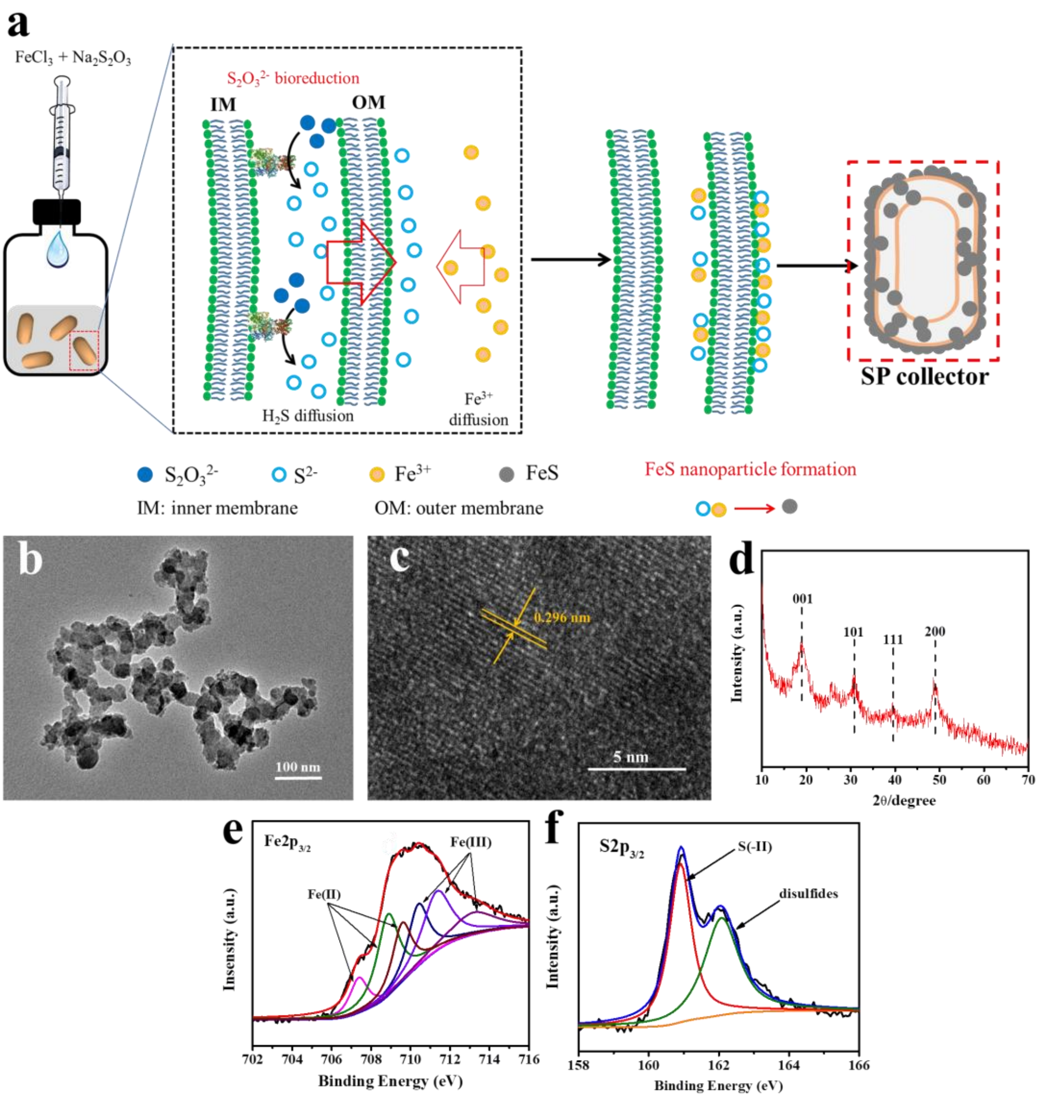

Supplementary Figure 3 | Assembly and characterization of SP collector on the SW cell. **(a)** The schematic illustration of the FeS biosynthesis and assembly on the bacterial periplasm and outer surface; **(b)** TEM, **(c)** HRTEM image and **(d)** XRD pattern and **(e)-(f)** XPS spectra of FeS nanoparticles collected from SW@SP. The indicated lattice spacing 0.296 nm in **(c)** was consistent with the (101) planes of mackinawite phase of FeS.

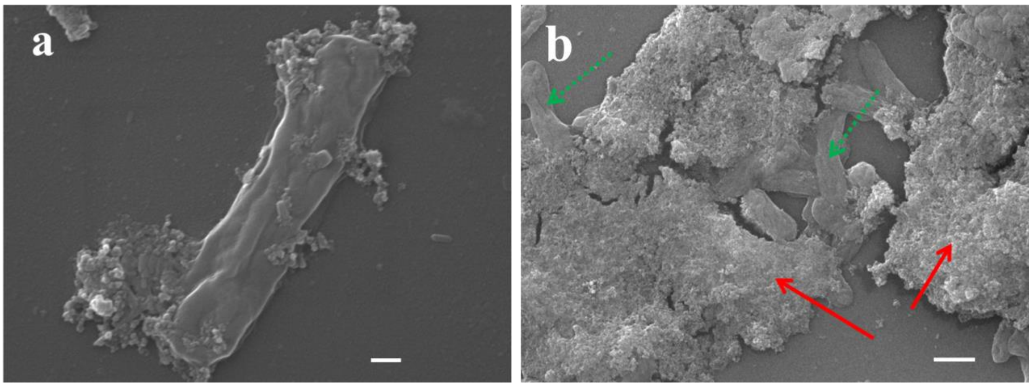

Supplementary Figure 4 | SEM images of FeS coated *Shewanella* cells under different Fe/S ratio. $FeCl_3$ was fixed at concentration of 0.1 mM, while $Na_2S_2O_3$ concentration was controlled at **(a)** 0.05 mM; **(b)** 0.5 mM. Red solid arrows indicated the large quantity of extracellular FeS, green dash arrow indicated the cell with smooth surface. Scale bars: (a) 200 nm; (b) 1 μm. According to these images, intact FeS nanoshell on cell surface was difficult to be assembled under these conditions.

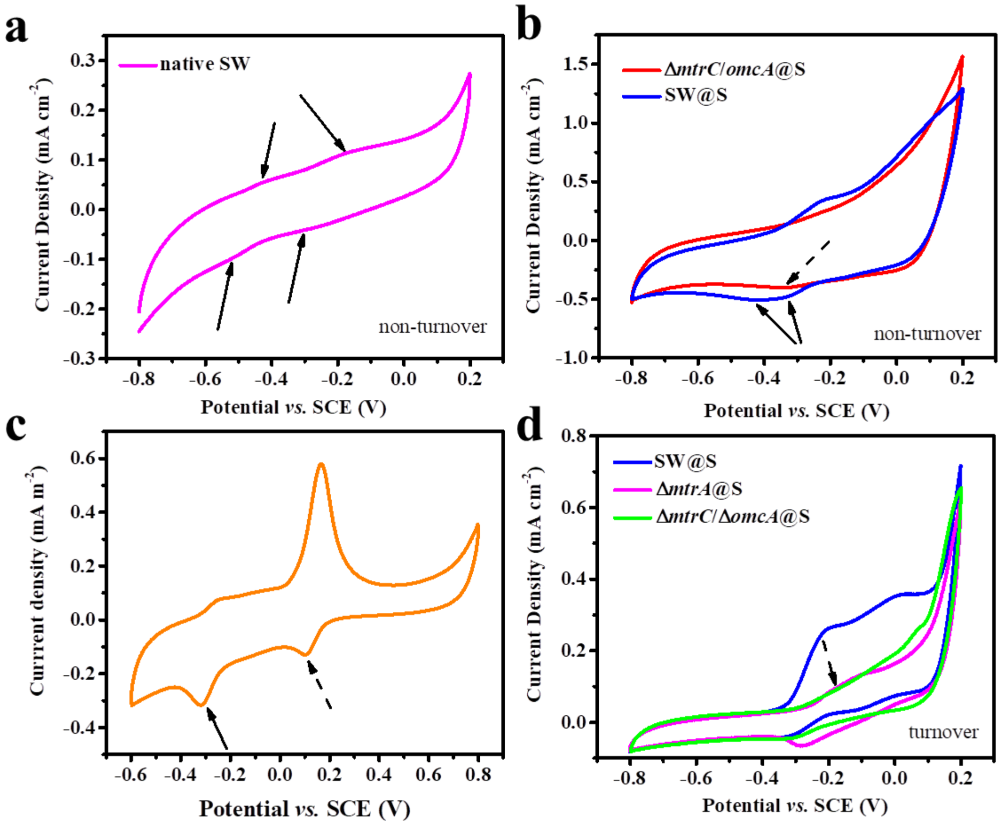

Supplementary Figure 5 | The CV curves. **(a)** Native SW cells under non-turnover conditions, scanning rate: 10 mV s^{-1}. Arrows indicates the peaks belonged to soluble flavin and OMC; **(b)** Polydopamine (PDA) assembled *S. oneidensis* cells (SW@S) colonized carbon felt electrode, the arrows indicated the reduction peak in the cathodic scanning. CV were conducted at non-turnover condition (nutrient depletion) with scanning rate of 10 mV s^{-1}; The baseline subtracted CV curve of SW@S cell (Fig. 4b) was obtained by directly subtracting CV curve of SW@S by Δ*mtrC*/*omcA*@S; **(c)** CV curve of PDA electrochemically deposited on the carbon felt electrode, dash and solid arrow indicated the relevant cathodic peaks . Deposit via: dopamine (4 mg mL^{-1}) in M9 salt medium, scanning rate of 50 mV s^{-1}, and data of 100th cycle was presented; **(d)** CV curves of SW@S, Δ*mtrA*@S and Δ*mtrC*/*omcA*@S colonized carbon felt electrode. The CV analyses were conducted at turnover condition with scanning rate 1 mV s^{-1}. Sigmoid shaped anodic curve with onset potential around -0.35 V indicated the use of flavins-bound OMC for continuous microbial discharge at SW@S interface. However, upon the disruption of MtrC/OmcA-MtrB-MtrA transmembrane electron conduit, the reduced current and positive drift of potential was observed, indicating reduced conductivity in the SW@S[3]. With the context of this work, it confirms that MtrC/OmcA-MtrB-MtrA transmembrane electron conduit was in responsibility for the electron transfer between cell metabolism and PDA.

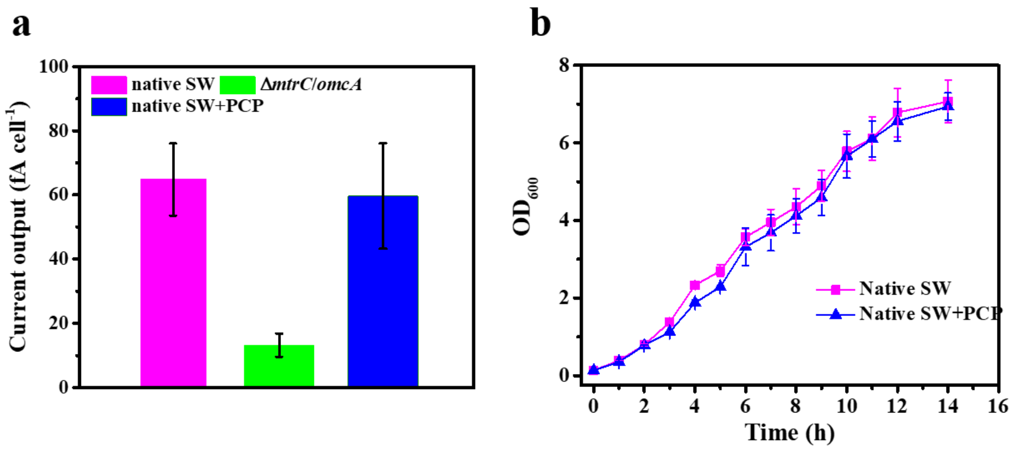

Supplementary Figure 6 | The current output and growth profile of SW cell in the presence of pentachlorophenol (PCP, 2 mg L^{-1}). **(a)** The current output; **(b)** The cell growth. The result showed that low concentration of PCP had negligible effect on the cell growth of *S. oneidensis* MR-1. Error bars represent standard error (s.e.) determined by three independent experiments.

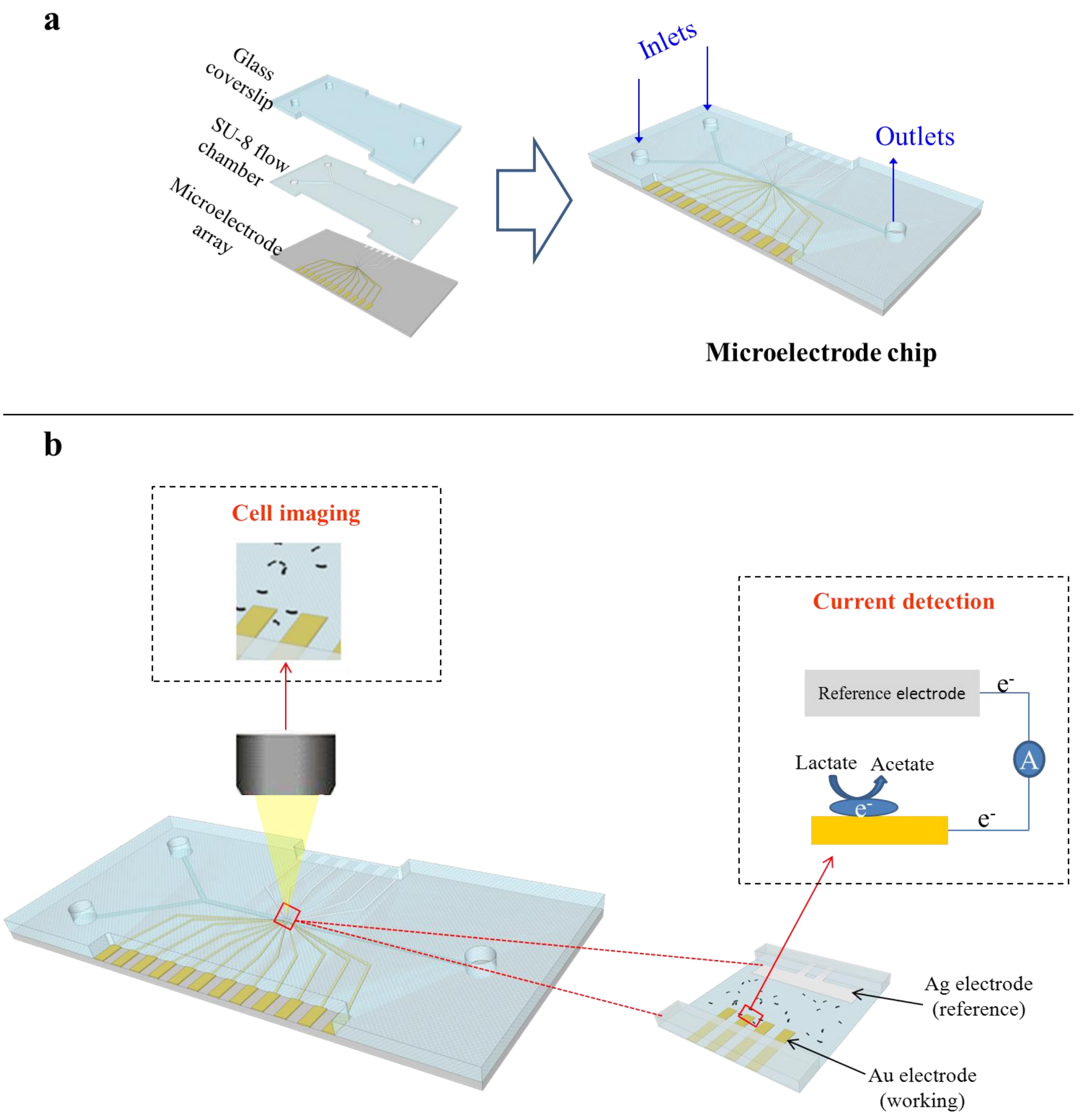

Supplementary Figure 7 | The design and operation of microelectrode chip. **(a)** Schematic for microelectrode chip fabrication and measurements. **(b)** Schematic of experimental set-up for simultaneous cell imaging with microscope and current measurement with electrochemical workstation. The black dots on the slides represent the bacterial cells landing on and around the microelectrode.

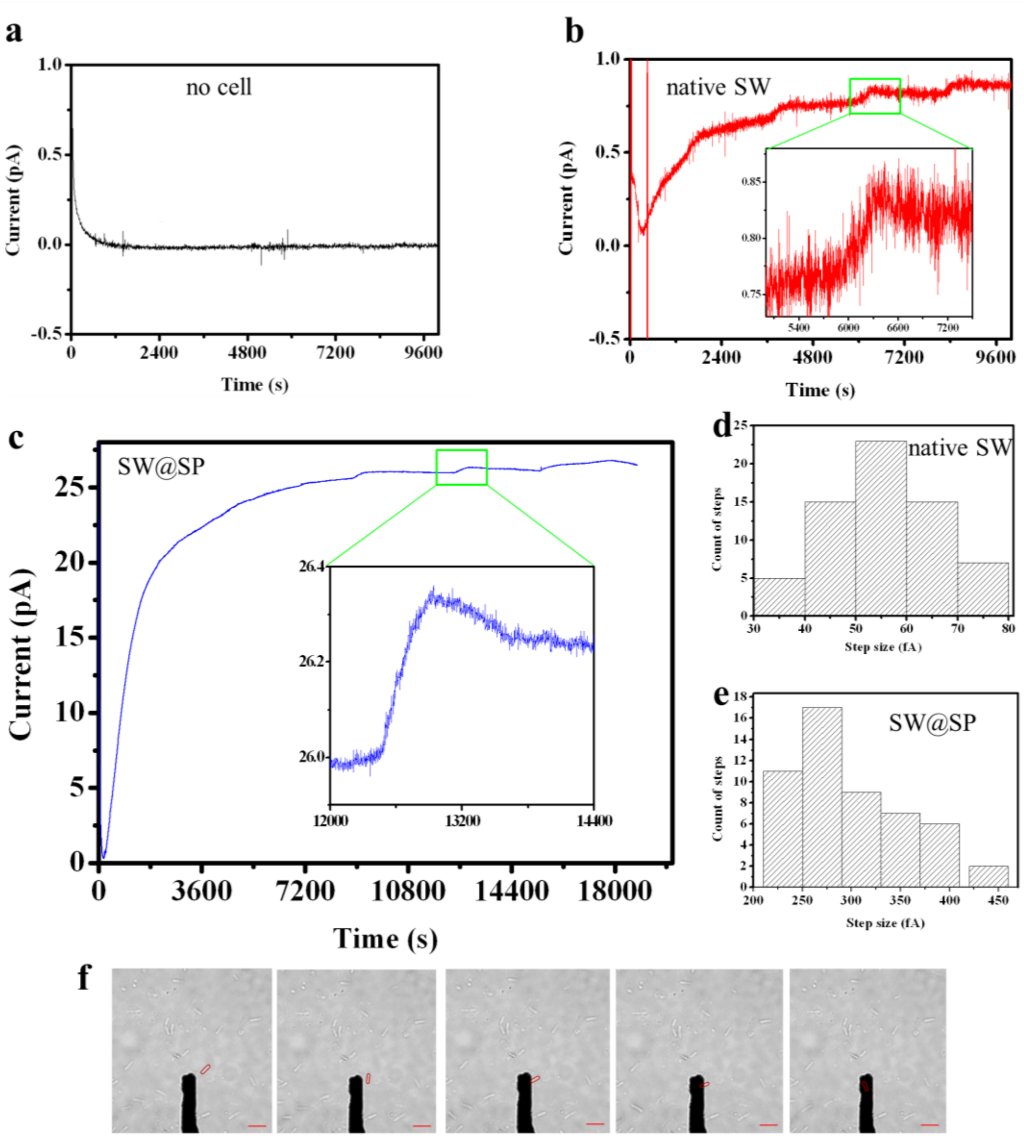

Supplementary Figure 8 | Analyses of stepwise current output with microelectrode chip. Short-circuit current recording from Au microelectrode in the bacterial medium **(a)** Without cell, **(b)** With native SW cell, or **(c)** With SW@SP cell. Insets of (b) and (c) indicate the enlarged image of the selected area of single cell induced stepwise current output as simultaneously tracked by microscope. **(d)-(e)** The amplitude distribution of the current steps recorded from more than 5 independent measurements. More than 50 counts of steps were applied for statistical analysis. **(f)** Typical tracking images of single cell landing onto the microelectrode. The cell that contact electrode concurrently with the stepwise current increase was highlighted with red circle line. The Au working electrode was poised at -0.1 V *vs.* SCE for all *i-t* measurements (the potential of the Ag reference electrode in the microelectrode chip was firstly calibrated with the SCE standard reference electrode). The scale bar in (f) is 5 μm.

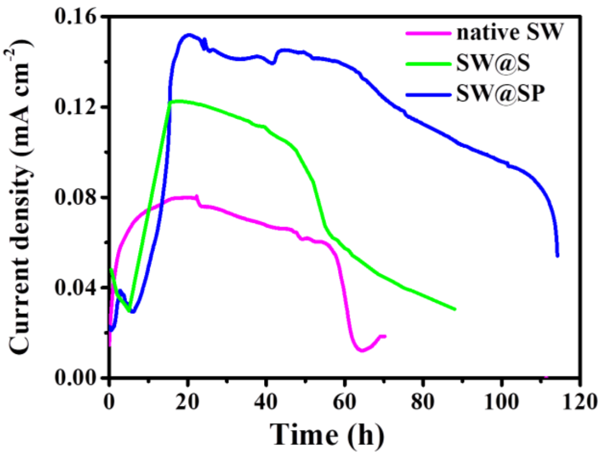

Supplementary Figure 9 | The time course of current output in MFC with native SW, SW@S and SW@SP cells, with constant external resistance of 2000 Ω. The electron recovery efficiency was estimated by dividing the total coulombs of electron released by theoretical value (nFCV, where n is the number of electron released for one molecular lactate consumption (n=4 in anaerobic inoculation, F is the Faraday constant, C is the consumed lactate concentration (initial 18 mM) and V is the liquid volume (30 mL). Notably, during the first 20 h, the consumed lactate in SW@SP system was mainly used for thiosulfate and Fe(III) reduction. Therefore electron recovery efficiency was estimated by subtracting the electron used for sulfur and Fe(III) reduction. Thiosulfate was completely reduced and XPS results indicated that the synthesized FeS nanoparticle had a chemical composition close to $FeS_{1.47}$. Considering totally 5 mM $FeCl_3$ and $N_2S_2O_3$ were added, 101 coulombs of charge was used for FeS production. In this situation, the electron recovery efficiency was 86.9%.

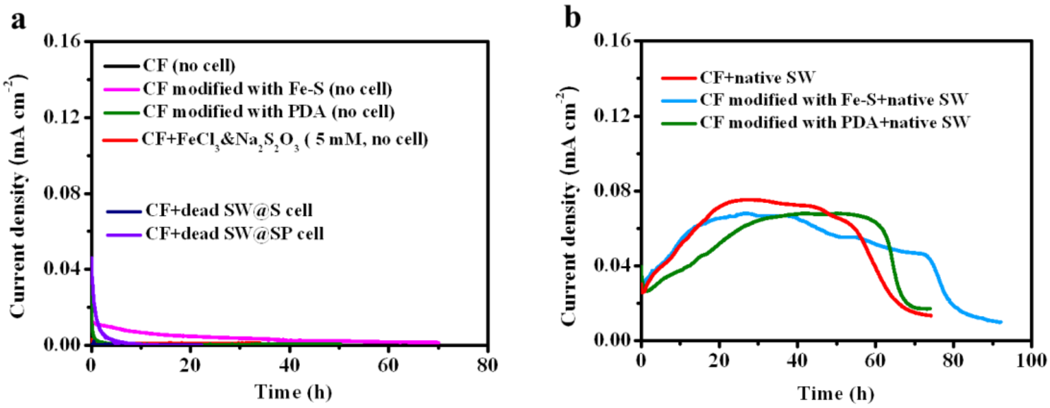

Supplementary Figure 10 | The time course current output of MFC under different conditions. **(a)** Current output of MFC operated without cell or dead cells. The SW@S or SW@SP cells were inactivated with isopropanol (70%) treatment for 10 min and the cell viability was checked by LIVE/DEAD staining to make sure no living cell remained. These results showed that the abiotic MFCs did not deliver significant current output as compared with MFCs with cells (Supplementary Fig. 9), which exclude the effect of these abiotic parameters. (b) Current output of native SW cells in MFC with different electrode. The material loading on electrode was strictly controlled by tuning the synthesis conditions (Supplementary Note 2) to make it is the same as that of SW@S or SW@SP MFC in Supplementary Fig. 9. These results indicated that the Fe-S nanoparticles or PDA modification on electrode surface only slightly affected the interfacial electron transfer. As compared with the effect of single cell electron collector (Supplementary Fig. 9), electrode only showed marginal effect on the interfacial electron transfer.

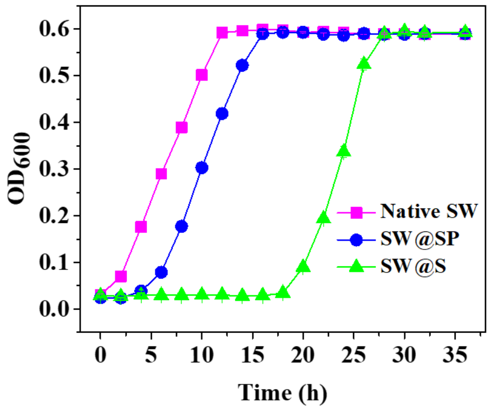

Supplementary Figure 11 | Growth curves of native SW, SW@SP and SW@S cells, respectively. Cells were cultivated in M9 salt medium with Wolfe mineral, Wolfe vitamin, fumarate (40 mM, soluble electron acceptor), and lactate (18 mM, sole carbon and energy source). All cells were anaerobically cultured under 30 °C with shaking (200 rpm).

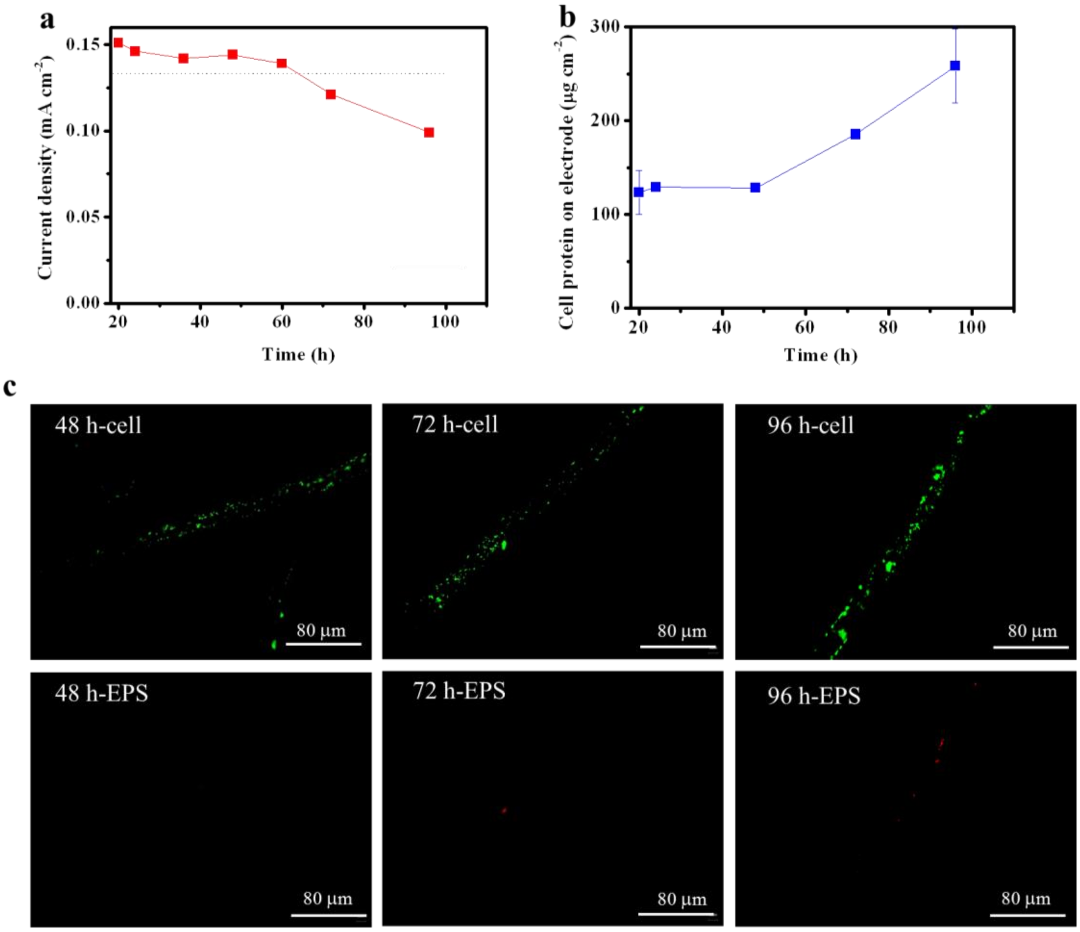

Supplementary Figure 12 | The mid-term performance and the biofilm development in BES inoculated with SW@SP cells. **(a)** The stability of current output from SW@SP biofilm in BES system. The SW@SP biofilm reached the highest current output at the 20th hour and maintained the high performance to the 60th hour. The current output was measured in BES system and all the conditions are same as that of Supplementary Fig. 9. The black line indicates 90% of the initial current density. **(b)** Growth of SW@SP cells on carbon felt electrode in BES system as indicated by cell protein content. Error bars represent standard error (s.e.) determined by three independent trials. **(c)** Fluorescence images of SW@SP biofilm on the fiber of carbon felt electrode. Biofilm was stained with SYTO 9 (for cell visualization, green) and Rhodamine labeled Concanavalin A (for EPS visualization[4], red). According to these results, cell growth of SW@SP biofilm on electrode was only started after 48 hours, while EPS production was only observed after 72 hours.

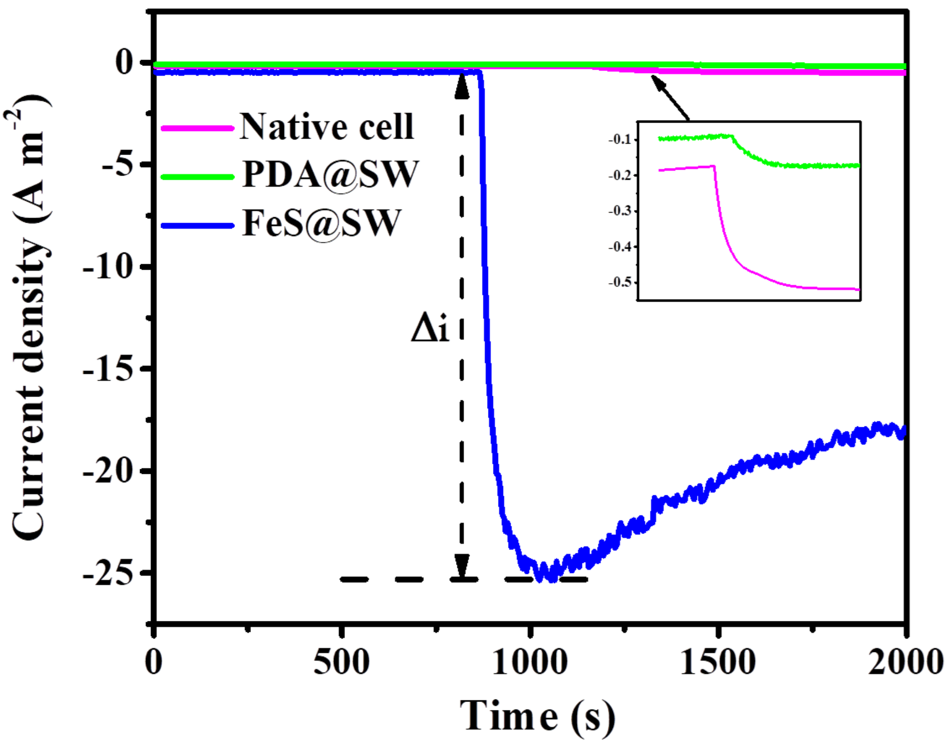

Supplementary Figure 13 | Current assimilation of native cells, SW@SP cells and sterilized SW@SP cells in the microbial electrosynthesis cell. The electrode potential was poised at -0.6 V and fumarate (20 mM) was added at steady discharge**.** The increased cathodic current (Δi) quantified the bioreduction rate of fumarate to succinate with the exogenous electron.

Supplementary Table 1: Performance comparison of high-performance MFC (power density >0.8 W m^{-2}, or CE>20%) inoculated with *S. oneidensis* MR-1.

Electrode	Engineering	MFC configuration	P_{max} (W m^{-2})	CE (%)	Ref.
PANi@GO@GF	Modification of GF with PPy and GO	D, 25 mL, $K_3Fe(CN)_6$	1.326	NM	5
rGO-CC	rGO hybridized biofilm on carbon electrode	D, 50 mL, $K_3Fe(CN)_6$	0.843	NM	6
Fe_3O_4@CNTs@GO	GO/CNTs/Fe_3O_4 foams	D, 50 mL, $K_3Fe(CN)_6$	1.711	NM	7
GO@TiO_2-Ni	GO/TiO_2 composite pasted to Nickel foam	D, 100 mL, $K_3Fe(CN)_6$	1.06	NM	8
CNT-GO-CF	Cell/CNT/rGO hybrid modified CF	D, 100 mL, $K_3Fe(CN)_6$	0.979	NM	9
rGO-CC	Strain genetic engineering and rGO modification	D, 50 mL, $K_3Fe(CN)_6$	2.63	NM	10
Pd@CC	Bio-Pd modified with carbon cloth	D, 150 mL, air cathode	0.605	40.5	11
CC	Genetic engineering	D, 140 mL, $K_3Fe(CN)_6$	0.163	21.7	12
GF	Miniature reactor with operation condition optimization	M, 1.2 mL, $K_3Fe(CN)_6$	NM	30	13
Carbon brush	Reactor configuration optimization	S, 325 mL, air	0.332	~32	14
CF	SW@SP	D, 30 mL, $K_3Fe(CN)_6$	3.21	86.9	This study

Abbreviations

PANi: polyaniline; GO: graphene oxide; rGO: reduced graphene oxide; GF: graphite felt; CC: carbon cloth; CF: carbon felt;
D: duel-chamber MFC; M: mini-MFC; S: single-chamber MFC;
NM: not mentioned.

Supplementary Table 2 | The respiration rate of individual cell with different electron acceptor. In most reports, the data was presented normalized to the unit total protein, and was recalculated here with the measured protein content of individual cell (1.53 $\times 10^{-13}$ g).

Strain	Electron acceptor	Electron transfer rate $\times 10^{6}$ e s^{-1} $cell^{-1}$	Cell physiology	Ref
S. oneidensis MR-1	Fe(III)-citrate, soluble	2.6	anaerobic, suspended	[15]
S. oneidensis MR-1	Fumarate, soluble	2.1	anaerobic, suspended	[16]
S. oneidensis MR-1	Nitrate, soluble	0.71	anaerobic, suspended	[17]
	Nitrite, soluble	0.96	anaerobic, suspended	
S. oneidensis MR-1	O_2, soluble	0.52	aerobic, suspended	[18]
S. oneidensis MR-1	O_2, soluble	0.28 ~ 2.8	aerobic, suspended	[19]
S. oneidensis MR-1	Fe-oxide, *insoluble*	0.047	anaerobic, suspended	[15]
S. oneidensis MR-1	Sudan dyes, *insoluble*	0.75	anaerobic, suspended	[20]
S. oneidensis MR-1	ITO electrode	0.62	BES , electrode colonized, single cell	[21]
		1.2	BES, electrode colonized, single cell	
S. oneidensis MR-1	Graphite rod electrode	1.3	MFC, electrode colonized, 2.0 $\times 10^{5}$ cell cm^{-2}	[22]
		0.59	MFC, electrode colonized, 8.8 $\times 10^{5}$ cell cm^{-2}	
S. oneidensis MR-1	Carbon felt electrode	2.5	MFC, electrode colonized, 2.5 $\times 10^{9}$ cell cm^{-2}	This work

Supplementary References

1. Bernsmann, F., Voegel, J.-C. & Ball, V. Different synthesis methods allow to tune the permeability and permselectivity of dopamine-melanin films to electrochemical probes. *Electrochim. Acta* **56**, 3914-3919 (2011).

2. Wang, R. et al. FeS_2 nanoparticles decorated graphene as microbial-fuel-cell anode achieving high power density. *Adv. Mater.* **30**, 1800618 (2018).

3. Torres, C. I. et al. Kinetic experiments for evaluating the Nernst-Monod model for anode-respiring bacteria (ARB) in a biofilm anode. *Environ. Sci. Technol.* **42**, 6593-6597 (2008).

4. Maeyama, R. et al. Confocal imaging of biofilm formation process using fluoroprobed *Escherichia coli* and fluoro-stained exopolysaccharide. *J. Biomed. Mater. Res. A* **70A**, 274-282 (2004).

5. Lv, Z. et al. One-step electrosynthesis of polypyrrole/graphene oxide composites for microbial fuel cell application. *Electrochim. Acta* **111**, 366-373 (2013).

6. Yong, Y.-C. et al. Highly active bidirectional electron transfer by a self-assembled electroactive reduced-graphene-oxide-hybridized biofilm. *Angew. Chem. Int. Ed.* **53**, 4480-4483 (2014)

7. Song, R.-B. et al. Bacteria-affinity 3D macroporous graphene/MWCNTs/Fe_3O_4 foams for high-performance microbial fuel cells. *ACS Appl. Mater. Interf.* **8**, 16170-16177 (2016).

8. Zhao, C.-e. et al. Nanostructured graphene/TiO_2 hybrids as high-performance anodes for microbial fuel cells. *Chem.- Eur. J.* **20**, 7091-7097 (2014).

9. Zhao, C.-e. et al. Hybrid conducting biofilm with built-in bacteria for high-performance microbial fuel cells. *Chemelectrochem* **2**, 654-658 (2015).

10. Lin, T. et al. Engineered *Shewanella oneidensis*-reduced graphene oxide biohybrid with enhanced biosynthesis and transport of flavins enabled a highest bioelectricity output in microbial fuel cells. *Nano Energy* **50**, 639-648 (2018).

11. Quan, X., Sun, B. & Xu, H. Anode decoration with biogenic Pd nanoparticles improved power generation in microbial fuel cells. *Electrochim. Acta* **182**, 815-820 (2015).

12. Li, F. et al. Modular engineering to increase intracellular NAD(H/+) promotes rate of extracellular electron transfer of *Shewanella oneidensis*. *Nat. Commun.* **9**, 3637

(2018).

13. Rosenbaum, M. A. et al. *Shewanella oneidensis* in a lactate-fed pure-culture and a glucose-fed co-culture with *Lactococcus lactis* with an electrode as electron acceptor. *Bioresource Technol.* **102**, 2623-2628 (2011).

14. Watson, V. J. & Logan, B. E. Power production in MFCs inoculated with *Shewanella oneidensis* MR-1 or mixed cultures. *Biotechnol. Bioeng.* **105**, 489-498 (2010).

15. von Canstein, H. et al. Secretion of flavins by *Shewanella species* and their role in extracellular electron transfer. *Appl. Environ. Microbiol.* **74**, 615-623 (2008).

16. Pinchuk, G. E. et al. Pyruvate and lactate metabolism by *Shewanella oneidensis* MR-1 under fermentation, oxygen limitation, and fumarate respiration conditions. *Appl. Environ. Microbiol.* **77**, 8234-8240 (2011).

17. Gao, H. et al. Reduction of nitrate in *Shewanella oneidensis* depends on atypical NAP and NRF systems with NapB as a preferred electron transport protein from CymA to NapA. *ISME J.* **3**, 966-976 (2009).

18. Pinchuk, G. E. et al. Constraint-based model of *Shewanella oneidensis* MR-1 metabolism: a tool for data analysis and hypothesis generation. *Plos Comput. Biol.* **6**, 1000822 (2010).

19. Riedel, T. E. et al. Oxygen consumption rates of bacteria under nutrient-limited conditions. *Appl. Environ. Microbiol.* **79**, 4921-4931 (2013).

20. Ji, Q. Y. et al. Removal of water-insoluble sudan dyes by *Shewanella oneidensis* MR-1. *Bioresource Technol.* **114**, 144-148 (2012).

21. Liu, H. et al. Electrochemical characterization of a single electricity-producing bacterial cell of *Shewanella* by using optical tweezers. *Angew. Chem. In. Ed.* **49**, 6596-6599 (2010).

22. McLean, J. S. et al. Quantification of electron transfer rates to a solid phase electron acceptor through the stages of biofilm formation from single cells to multicellular communities. *Environ. Sci. Technol.* **44**, 2721-2727 (2010).
